# Plant-Derived Zein as an Alternative to Animal-Derived Gelatin for Use as a Tissue Engineering Scaffold

**DOI:** 10.1002/anbr.202300104

**Published:** 2023-12-22

**Authors:** Apurva Limaye, Venkatesan Perumal, Courtney M. Karner, Treena Livingston Arinzeh

**Affiliations:** Department of Biomedical Engineering, New Jersey Institute of Technology, Newark, NJ 07102, USA; Department of Biomedical Engineering, Columbia University, 3960 Broadway, New York, NY 10027, USA; Department of Biomedical Engineering, New Jersey Institute of Technology, Newark, NJ 07102, USA; Department of Internal Medicine, Charles and Jane Pak Center for Mineral Metabolism and Clinical Research, University of Texas Southwestern Medical Center, Dallas, TX 75390, USA; Department of Biomedical Engineering, Columbia University, 3960 Broadway, New York, NY 10027, USA

**Keywords:** electrospinning, gelatin, mesenchymal stem cells, tissue engineering, zein

## Abstract

Natural biomaterials are commonly used as tissue engineering scaffolds due to their biocompatibility and biodegradability. Plant-derived materials have also gained significant interest due to their abundance and as a sustainable resource. This study evaluates the corn-derived protein zein as a plant-derived substitute for animal-derived gelatin, which is widely used for its favorable cell adhesion properties. Limited studies exist evaluating pure zein for tissue engineering. Herein, fibrous zein scaffolds are evaluated in vitro for cell adhesion, growth, and infiltration into the scaffold in comparison to gelatin scaffolds and are further studied in a subcutaneous model in vivo. Human mesenchymal stem cells (MSCs) on zein scaffolds express focal adhesion kinase and integrins such as *α*_v_*β*_3_, *α*_4_, and *β*_1_ similar to gelatin scaffolds. MSCs also infiltrate zein scaffolds with a greater penetration depth than cells on gelatin scaffolds. Cells loaded onto zein scaffolds in vivo show higher cell proliferation and CD31 expression, as an indicator of blood vessel formation. Findings also demonstrate the capability of zein scaffolds to maintain the multipotent capability of MSCs. Overall, findings demonstrate plant-derived zein may be a suitable alternative to the animalderived gelatin and demonstrates zein’s potential as a scaffold for tissue engineering.

## 1. Introduction

Tissue engineering approaches can utilize scaffolds to provide support for cells by enabling cells to adhere, grow, and deposit extracellular matrix.^[1]^ Scaffolds composed of natural biomaterials, such as proteins and polysaccharides from plant and animal sources, are investigated due to advantages in biocompatibility, biodegradability, and contribution to cellular function.^[2]^ Plant-derived materials have also gained attention due to their abundance and as a sustainable resource.^[3]^ Zein, a storage protein from corn, can be promising for tissue engineering applications. Zein is biocompatible and can be biodegraded enzymatically by physiological enzymes like collagenase.^[4]^ It has also exhibited favorable properties for cell adhesion and growth and antimicrobial properties.^[5]^ Zein contains hydrophobic amino acids such as leucine, proline, and alanine making it suitable for coatings and as microspheres for drug delivery. Zein is generally recognized as safe by the United States Food and Drug Administration (FDA) and is approved for use as a coating for pharmaceuticals.^[6]^ Studies have demonstrated the potential of zein in combination with ceramics and synthetic or natural polymers for bone tissue engineering.^[7,8]^ Few studies exist using zein alone as a porous substrate for tissue engineering^[9,10]^ due to its limited hydrolytic stability.^[11,12]^ Studies have shown that cells attach to zein, which may be via transglutaminase secreted by cells and/or the tripeptide leucine–aspartic acid–valine (LDV), which is an integrin-binding motif found in zein and extracellular matrix proteins including fibronectin.^[13]^ However, an understanding of the mechanism of cell adhesion to zein is still needed.

**Figure 1.**
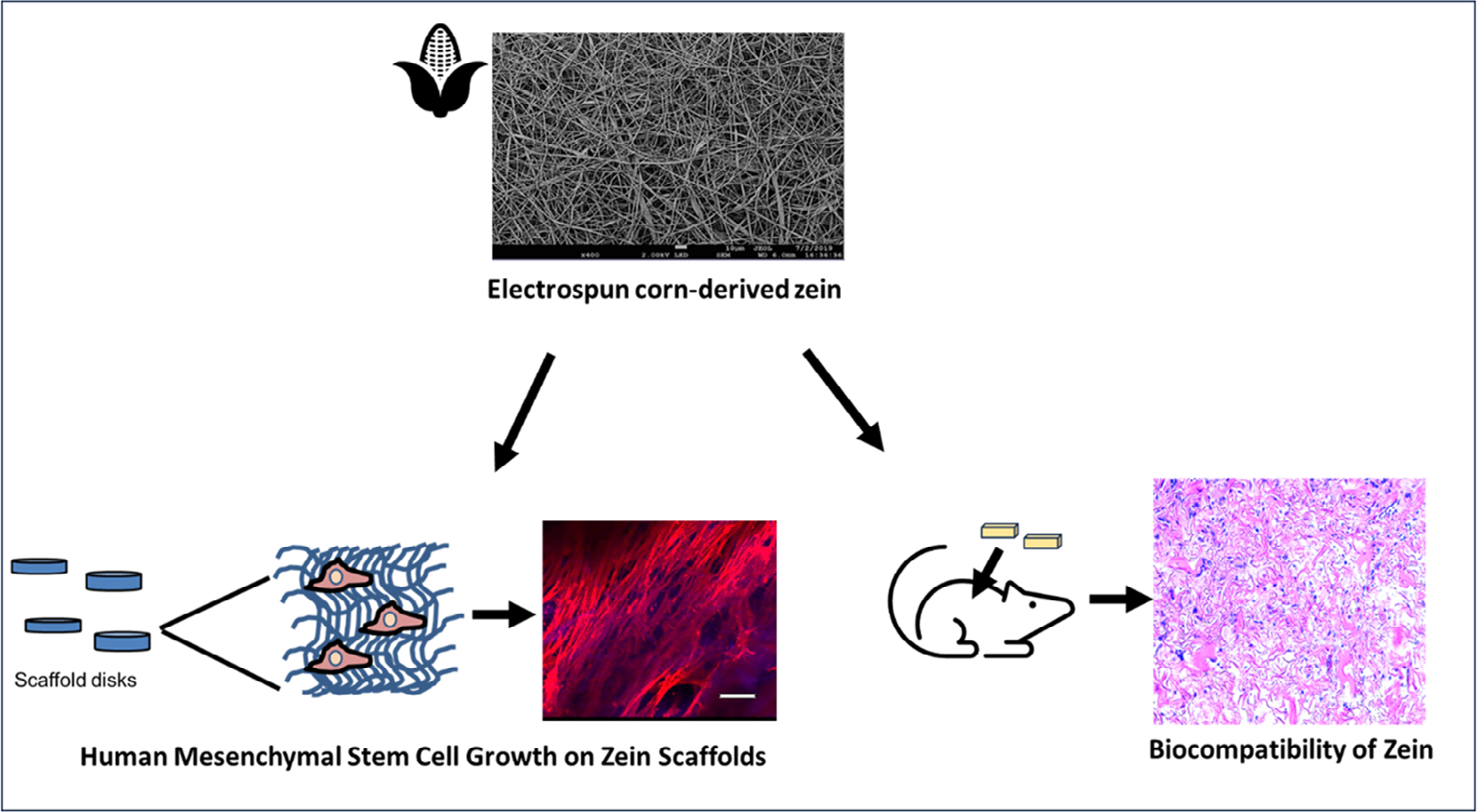
Schematic illustration of the electrospun zein scaffold and its evaluation for use as a tissue engineering scaffold. Zein scaffolds supported human MSC growth, adhesion, and infiltration into the scaffold. Comparisons were performed with gelatin scaffolds. The zein scaffolds also supported the maintenance of MSC multipotency. The biocompatibility of the zein scaffolds with or without MSCs was also determined in a subcutaneous model. Findings demonstrate the potential of zein as a tissue engineering scaffold.

In this study, zein was evaluated for its potential as a tissue engineering scaffold (Figure 1). Zein scaffolds were fabricated using the electrospinning technique to form a fibrous structure to more closely mimic the native extracellular matrix.^[1,2,14]^ To form hydrolytically stable scaffolds, our group has shown that fibrous zein can be cross-linked using trimethylolpropane triglycidyl ether (TMPGE) and maintain its fiber morphology after 30 days in aqueous environment.^[15]^ Here, we evaluated cross-linked fibrous zein scaffolds for supporting human mesenchymal stem cell (MSC) adhesion, growth, and infiltration into the scaffold in vitro, which has not been previously studied. Comparisons were made with animal-derived gelatin, which is well-established as favorable for cell adhesion and widely used in tissue engineering.^[16]^ For the first time, focal adhesion kinase (FAK) and integrin expression of MSCs on zein scaffolds were investigated as well as cell infiltration into the scaffolds, which is necessary for tissue integration.^[17]^ To establish the biocompatibility of the zein scaffold and its potential as a carrier for MSCs, zein scaffolds with or without MSCs were evaluated in vivo in a subcutaneous model. Cell proliferation and vascularization were determined. In addition, to establish the zein scaffold as a potentially viable substrate for the expansion of MSCs, the maintenance of MSC multipotency was determined on the zein scaffolds in vitro. This study evaluated zein, a plant-derived protein, as an alternative to the animal-derived protein gelatin and demonstrated zein’s potential as a scaffold for tissue engineering.

## 2. Results

### 2.1. Characterization of Scaffolds

**Figure 2.**
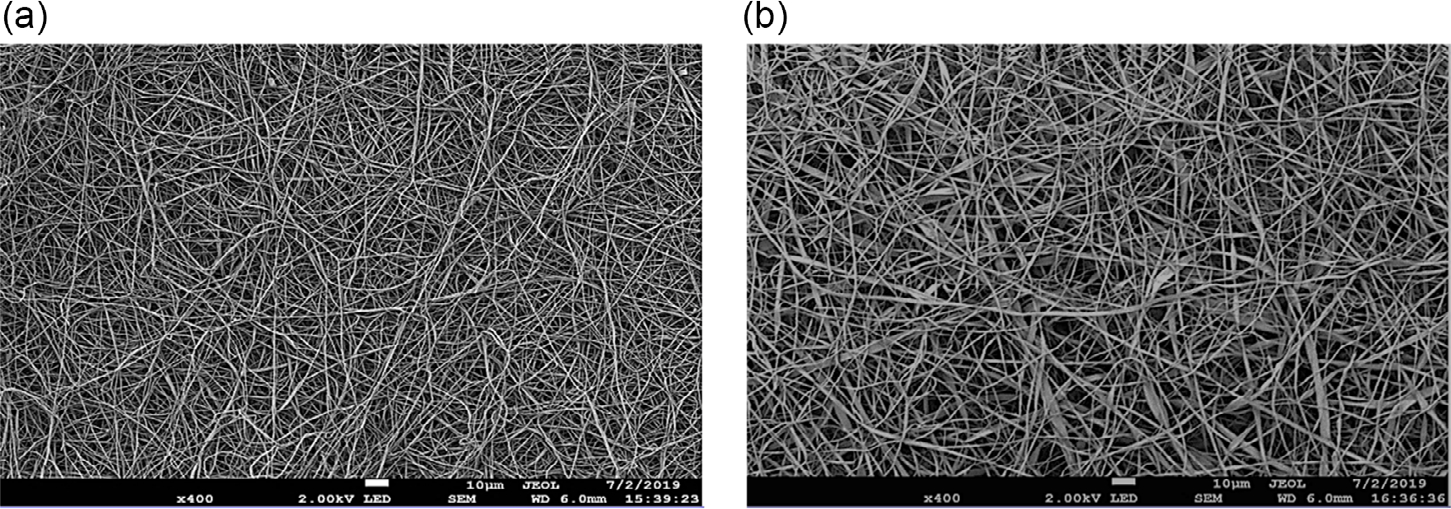
SEM images of cross-linked a) gelatin and b) zein. 400× magnification, scale bar is 10 μm.

Cross-linked gelatin (Figure 2a) and zein (Figure 2b) scaffolds were characterized by scanning electron microscopy (SEM). They had a uniform fibrous morphology, having similar fiber diameters of ≈1–2 μm and interfiber spacing of approx. 25 μm. Both scaffolds also had a Young’s modulus of ≈200 kPa., as determined by tensile testing (Table 1).

**Figure 3.**
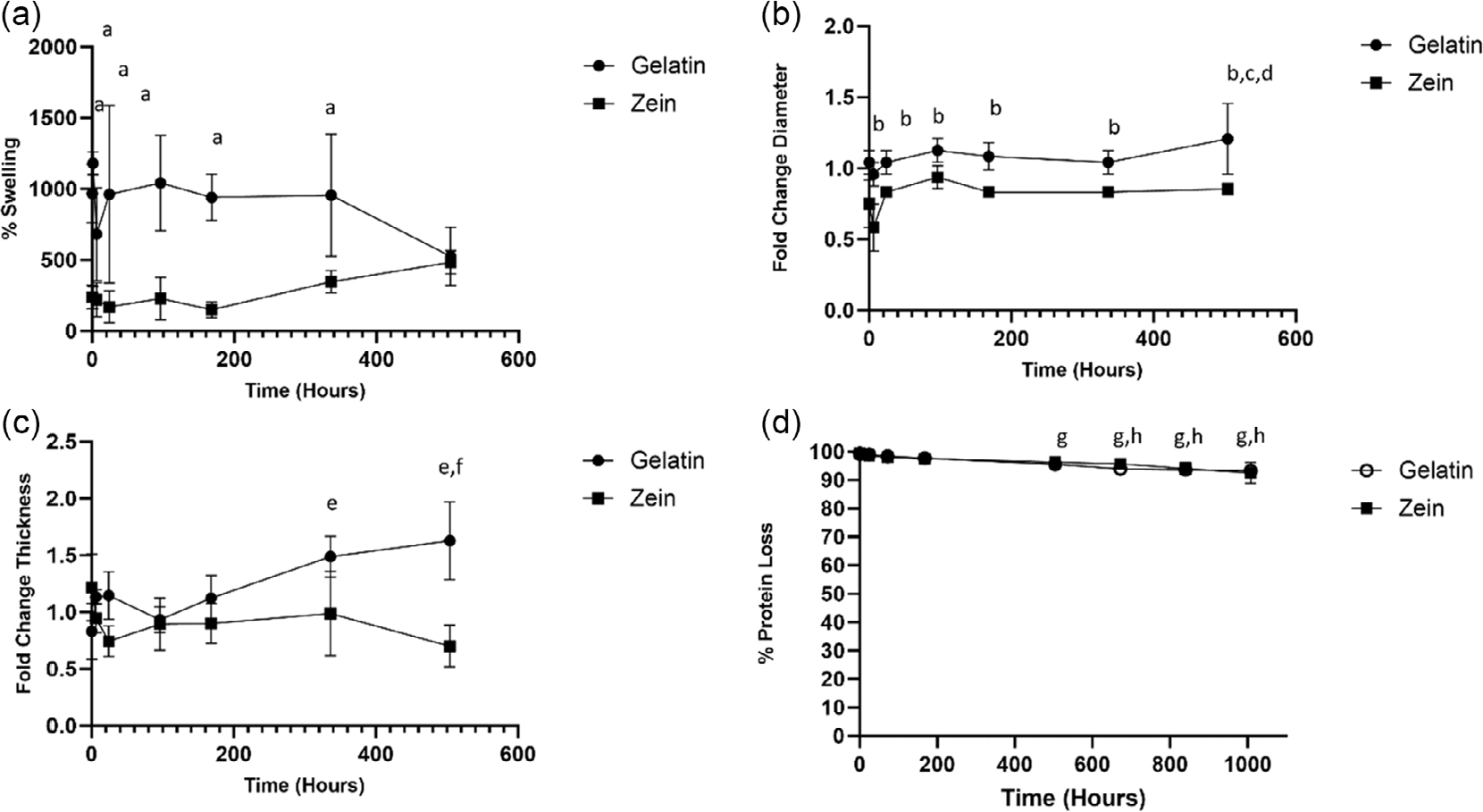
Hydrolytic stability of cross-linked gelatin and zein scaffolds. a) Percent swelling. b) Diameter changes reported as fold change to initial diameter. c) Thickness changes reported as fold change to initial thickness. d) Percent loss of protein. ^a^*p* < 0.05 for gelatin as compared to zein at 0.16, 1, 6, 24, 96, 168 h. ^b^*p* < 0.05 for zein at 0.16 and 6 h, ^c^*p* < 0.05 for gelatin at 504 h as compared to zein at 168, 336, 504 h, ^d^*p* < 0.05 for gelatin at 96 h compared to gelatin at 504 h. ^e^*p* < 0.05 for gelatin at 336 and 504 h compared to gelatin at 0.16 h and zein at 24, 96, 168, 336, and 504 h. ^f^*p* < 0.05 for gelatin at 504 h compared to zein at 6 h and gelatin at 96 h. ^g^*p* < 0.05 for gelatin and zein at 0.167,1, 24 h. ^h^*p* < 0.05 for gelatin and zein at 72 and 168 h.

Hydrolytic stability studies of cross-linked scaffolds were performed. Zein scaffolds had significantly less swelling than gelatin scaffolds at all time points, except at 504 h (Figure 3a). No statistical differences in percent swelling were detected after 0.16 h for either gelatin or zein over time except for 1 h of gelatin compared to 504 h of gelatin. Zein scaffolds also had a smaller diameter after hydration as compared to gelatin scaffolds (Figure 3b) where both scaffolds demonstrated no changes in diameter over time. The thickness of the scaffolds for both gelatin and zein was not statistically different at the initial time points (Figure 3c). Statistically significant differences in thickness for gelatin compared to zein were observed at 336 and 504 h where zein was significantly lower than gelatin. For protein loss, both scaffolds exhibited similar percent protein loss over time, with a percent protein loss of 7.4% ± 3.6% (zein) and 6.7% ± 1.0% (gelatin) by 1008 h (42 days) (Figure 3d).

### 2.2. Cell Growth and Viability

**Figure 4.**
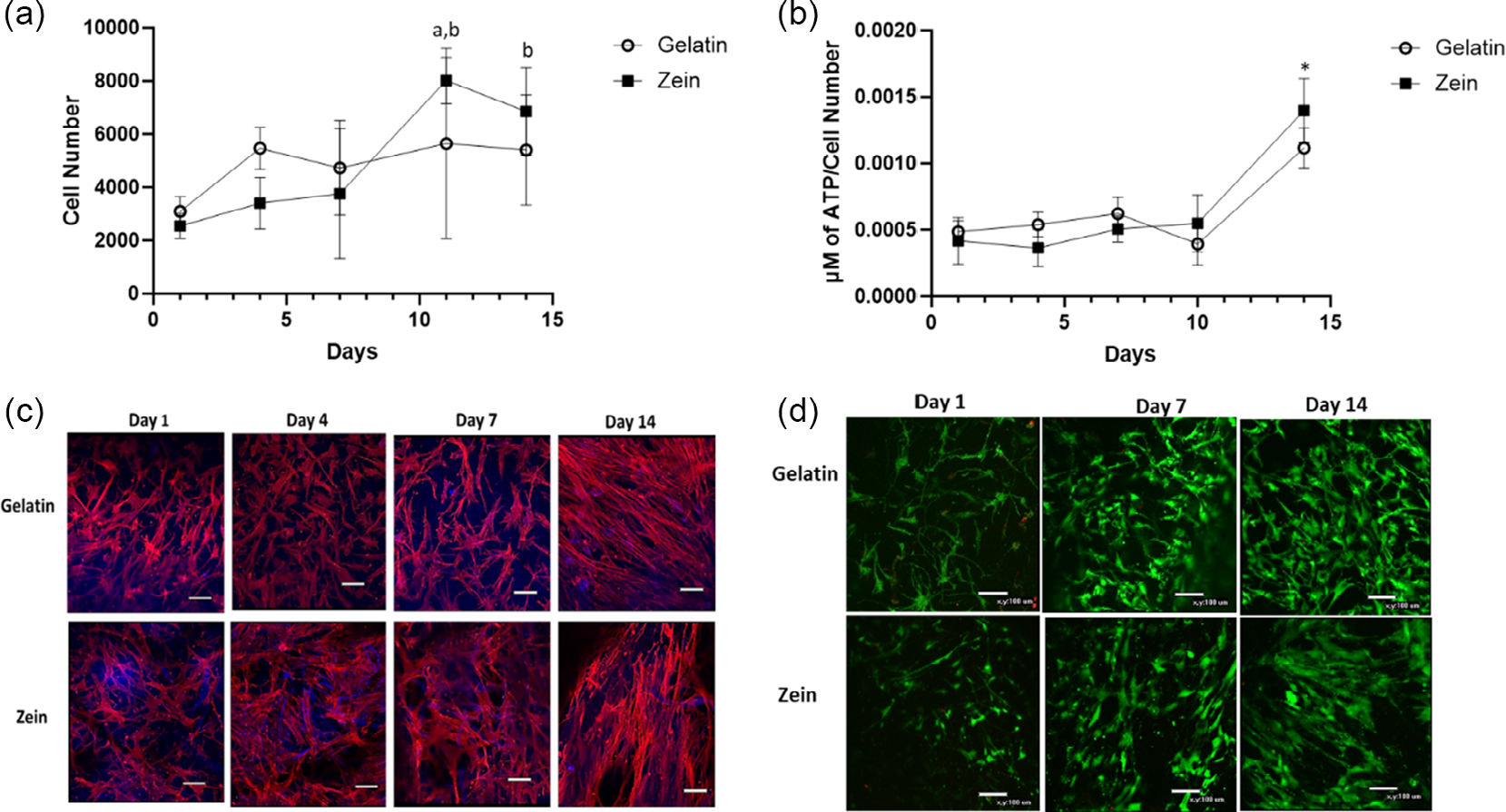
a) Average cell number for cells on gelatin and zein scaffolds over 14 days. b) μM of ATP normalized to average cell number for gelatin and zein scaffolds over 14 days c) Confocal imaging of MSCs seeded on scaffolds. Actin (red) stained with phalloidin and fibers (blue). Images were taken at 20× magnification. d) Live (green)/dead (red) staining of MSCs seeded on gelatin and zein scaffolds over 14 days. ^a^*p* < 0.05 was significantly different from the gelatin group on day 1 and from the zein group on days 1, 4, and 7. ^b^*p* < 0.05 was significantly different from the zein group on day 1. *p < 0.05 compared to all other groups for both zein and gelatin groups.

Cell number increased over time for zein scaffolds (Figure 4a). The cell number for cells on zein at day 11 was significantly higher than on days 1, 4, and 7 and the cell number on zein at day 14 was significantly greater than day 1. No significant differences in cell number were determined for the gelatin group over time. ATP measurements as an indicator of cell viability showed both scaffold groups had a significant increase in ATP at day 14 as compared to all other time points (Figure 4b). As shown in Figure 4c, cells attached to both scaffolds as indicated by an intense stain for actin filaments and cells had a spindle-like morphology and spreading. Cell viability was also confirmed by live/dead staining (Figure 4d). Mostly live cells were present on both scaffolds over 14 days.

### 2.3. Cell Adhesion in Serum-Free Conditions and Infiltration into the Scaffold

**Figure 5.**
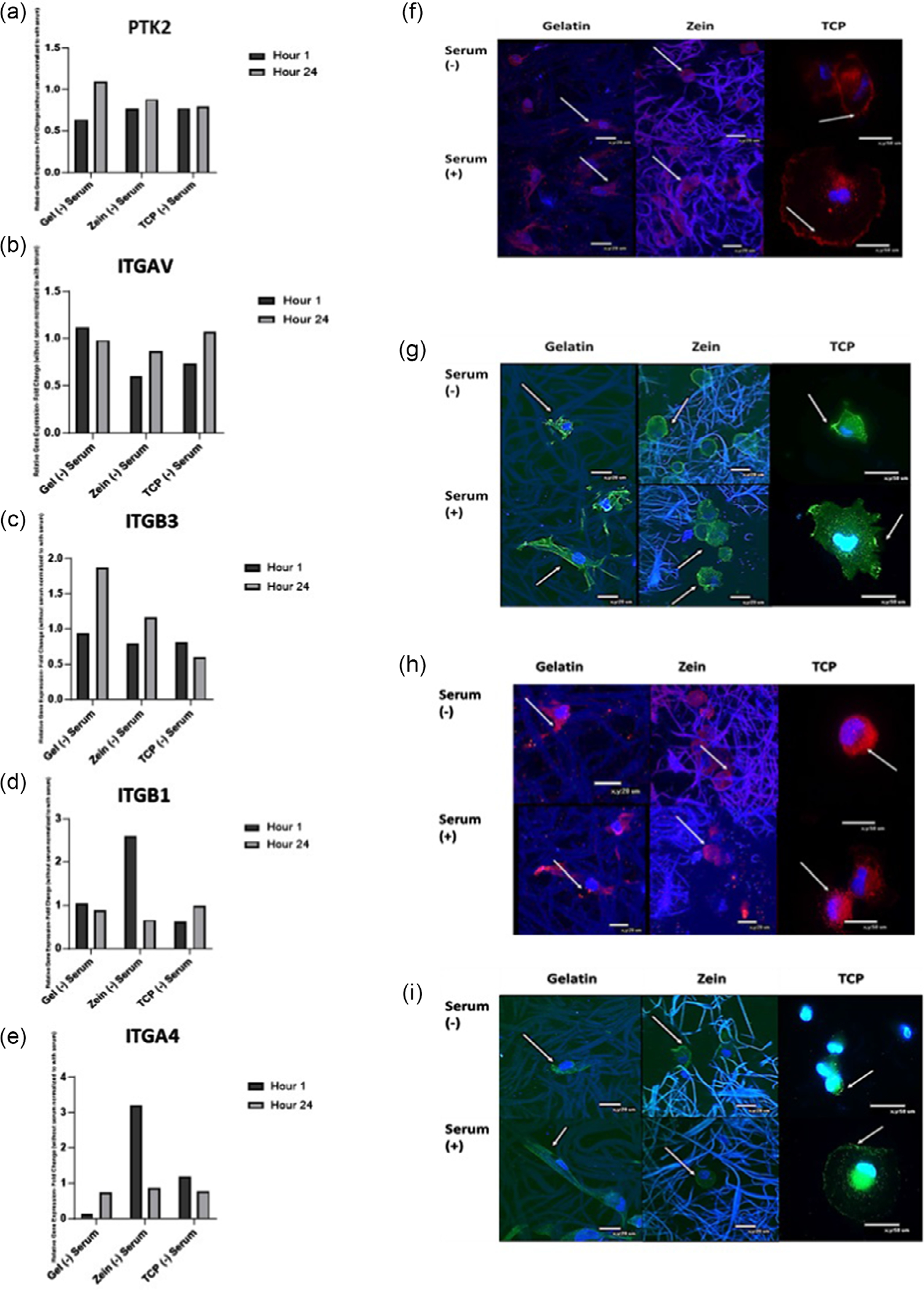
Relative gene expression for a) PTK2, b) ITGAV (*αν*), c) ITGB3 (*β*_3_), d) ITGB1 (*β*_1_), and e) ITGA4 (*α*_4_) at 1 and 24 h where the without serum group is normalized to the with serum group. Values are the mean of samples run in triplicate. f) FAK immunostaining for gelatin, zein, and TCP groups at 1 h. Nucleus was stained in blue and focal adhesion kinase in red. g) *ανβ*_3_ immunostaining. Nucleus was stained in blue and *ανβ*_3_ in green.h) *β*_1_ immunostaining for gelatin, zein, and TCP at 1 h with or without serum. Nucleus was stained in blue and *β*_1_ in red. i) *α*_4_ immunostaining. Nucleus was stained in blue and *α*_4_ in green. Scale bar: 20 μm for scaffolds and 50 μm for TCP.

Cell adhesion was further evaluated for cells on gelatin and zein scaffolds by examining adhesion in serum-free conditions and compared to tissue culture polystyrene (TCP) plates as a control. In serum-free and serum-containing conditions, similar cell numbers were obtained with or without serum at 1 h (Figure S1a, Supporting Information). Cells on gelatin and zein scaffolds exhibited a more spread morphology at 1 h in conditions with serum compared to a more rounded morphology in conditions without serum (Figure S1b, Supporting Information). By 24 h, cell number for gelatin and zein groups with serum was higher than without serum conditions (Figure S1c, Supporting Information). However, cells on gelatin and zein scaffolds with or without serum had a spread morphology at 24 h as demonstrated by actin staining (Figure S1d, Supporting Information). Relative gene expression for adhesion markers, where the expression for groups without serum was normalized to groups with serum, is shown in Figure 5. Gene expression for protein kinase 2 (PTK2), the gene that encodes FAK, was comparable across groups at 1 and at 24 h, the gelatin group had higher expression than the zein and TCP groups (Figure 5a). The presence of FAK was detected by immunostaining for the scaffold and TCP groups in both conditions at 1 and 24 h (Figure 5f and S2a, Supporting Information). Gene expression for α_V_*β*_3_, an integrin expressed when cells attach to the arginine–glycine–aspartic acid (RGD) amino acid sequence found in gelatin and fibronectin,^[16]^ was evaluated using ITGAV, the gene that encodes for *α*_V_ and ITGB3, the gene that encodes for *β*_3_. ITGAV gene expression was highest for the gelatin group as compared to zein and TCP groups at 1 h (Figure 5b). ITGB3 gene expression for the gelatin group was the highest as compared to the other groups at 24 h (Figure 4c). *α*_V_*β*_3_ was observed by immunostaining at 1 and 24 h for all groups with or without serum (Figure 5 g and S2b, Supporting Information). *α*_4_*β*_1_ is an integrin that is expressed when binding to the LDV amino acid sequence that is found in zein and fibronectin.^[13]^ Gene expression for ITGB1, the gene that encodes for *β*_1,_ was highest for the zein group at 1 h (Figure 5d). *β*_1_ integrin was observed by immunostaining for all groups (Figure 5h and S2c, Supporting Information). ITGA4, the gene that encodes for *α*_4_, had the highest expression for the zein group at 1 h (Figure 5e). Immunostaining demonstrated the presence of *α*_4_ in all groups (Figure 5i and S2d, Supporting Information).

**Figure 6.**
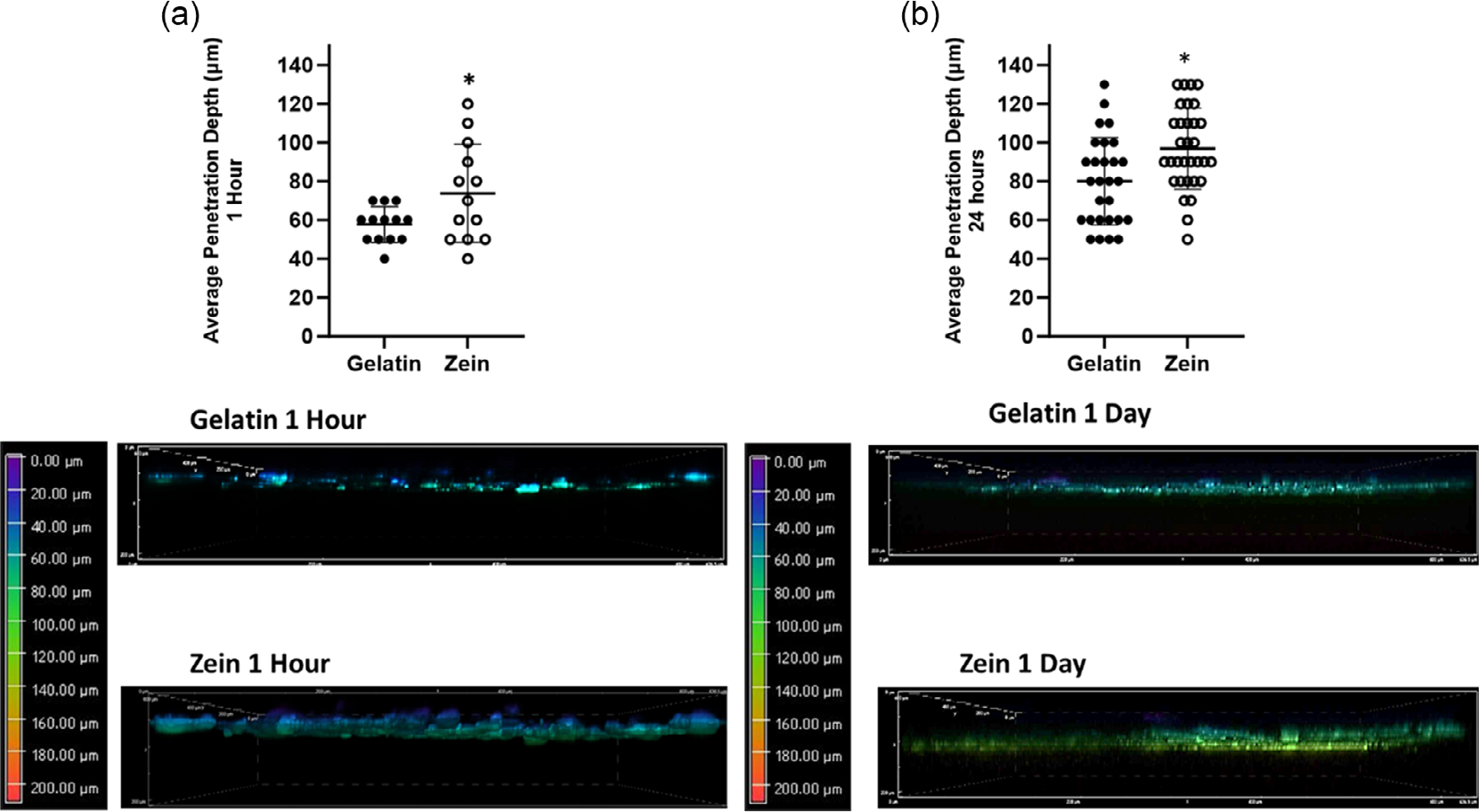
Average penetration depth of gelatin and zein groups at a) 1 h and b) 24 h. *p < 0.05 for zein compared to gelatin. Representative images of 3D volume projections and orthogonal sliced view of penetration depth.

Cell infiltration into the scaffold was evaluated at 1 h (Figure 6a) and 1 day (Figure 6b). Significantly larger penetration depth was determined for cells on zein scaffolds compared to gelatin scaffolds at 1 h and 1 day.

### 2.4. Subcutaneous Implantation of Zein Scaffolds

**Figure 7.**
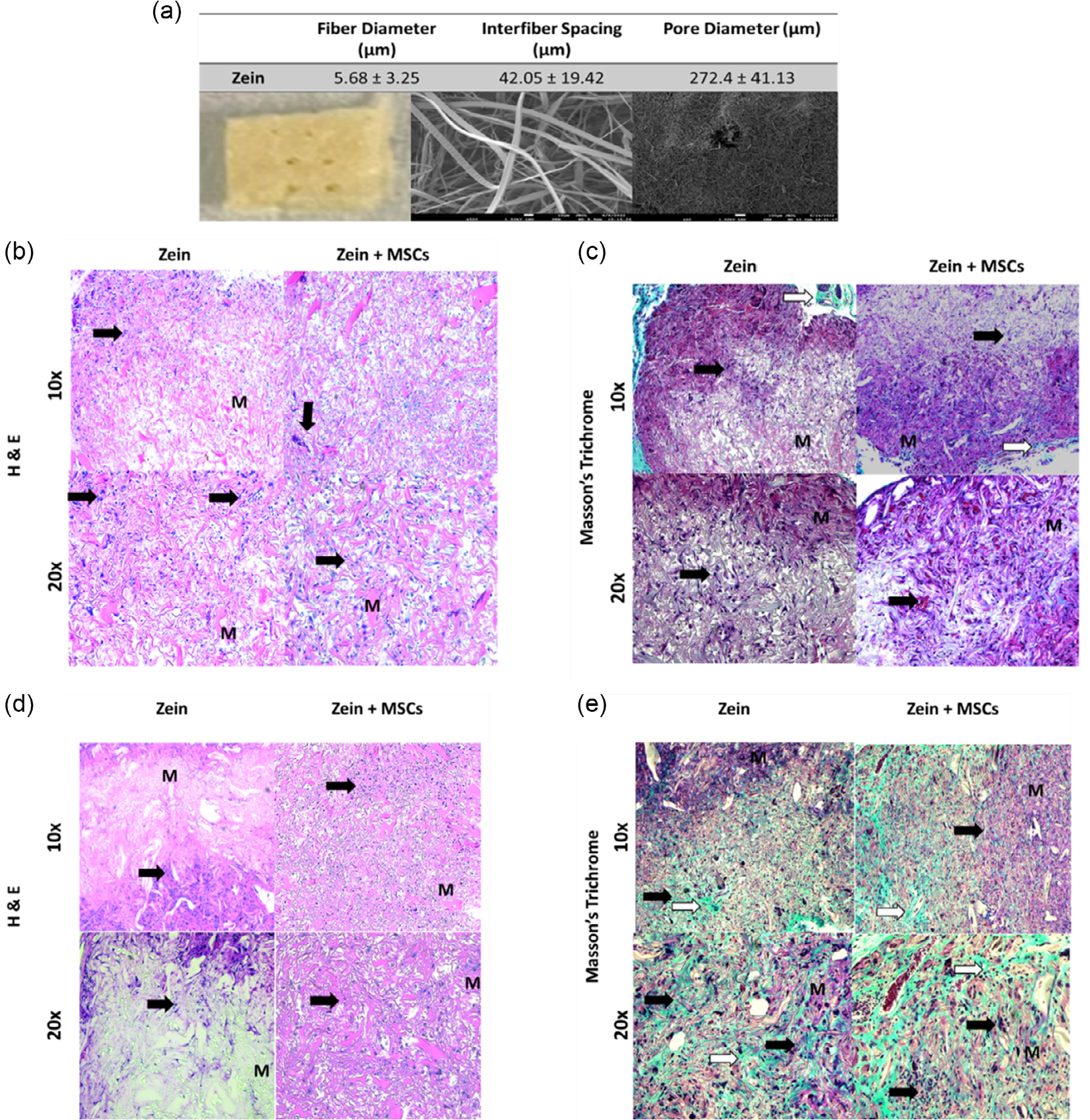
a) Gross and SEM images of zein implants for subcutaneous studies. Representative histological micrographs of cell-free or cell-loaded zein implants at 2 weeks postimplantation. b) H&E and c) Masson’s trichrome staining. Representative histological micrographs of cell-free or cell-loaded implants at 6 weeks postimplantation d) H & E and e) Masson’s trichrome staining. M represents scaffold material. Black arrows show cells in the implant. White arrows show the loose connective tissue, stained blue. Magnification 10× and 20×.

Zein scaffolds loaded with or without MSCs were further evaluated subcutaneously in mice as an initial study to investigate biocompatibility. The cross-linked zein scaffolds for implantation had an average fiber diameter and interfiber spacing of ≈5 and 42 μm, respectively (Figure 7a). Additional pores, having an average diameter of 272 μm, were created in the scaffold to promote cell infiltration and blood vessel formation. Cells were present throughout both cell-loaded and cell-free scaffolds (Figure 7b) with the appearance of loose connective tissue (stained blue) in the periphery of the implants as shown using Masson’s trichrome stain (Figure 7c). By 6 weeks, cells and connective tissue appeared to be greater as compared to 2 weeks (Figure 7d,e). Additionally, no adverse immune response was seen based on hemoxytolin and eosin (H & E) staining. The fibrous structure of the zein scaffold was still observed at 6 weeks postimplantation (Figure 7d).

**Figure 8.**
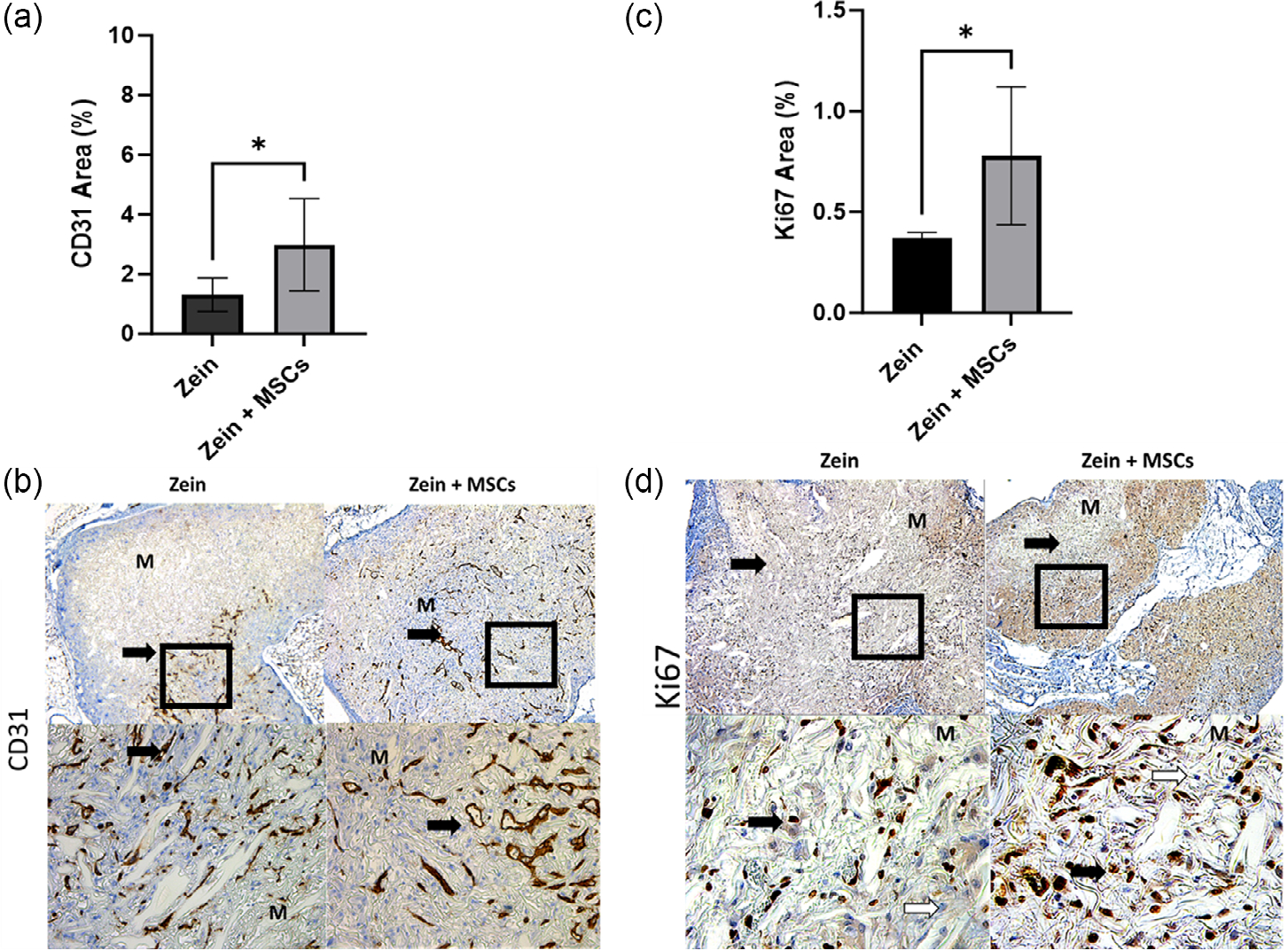
a) Quantification of CD31 and b) representative histological micrographs of CD31. c) Quantification of Ki67 and d) representative histological micrographs of Ki67. Histology is at 2 weeks postimplantation. M represents scaffold material. Black arrows show positive CD31 and Ki67. The squares represent the area viewed at 20x. Magnification 4 and 20. *p < 0.05 was significantly different for zein compared to zein + MSCs.

Immunostaining for mouse-specific CD31, which is a marker for vascularization/blood vessels, was detected at 2 weeks with greater CD31 for zein implants loaded with cells compared to cell-free implants (Figure 8a). Representative images of CD31 staining showed the formation of blood vessels in the scaffolds (Figure 8b). Ki67, a proliferation marker, was observed where cell-loaded zein implants had greater Ki67 than cell-free implants (Figure 8c,d).

### 2.5. Multipotent Capability of MSCs on Zein Scaffolds

**Figure 9.**
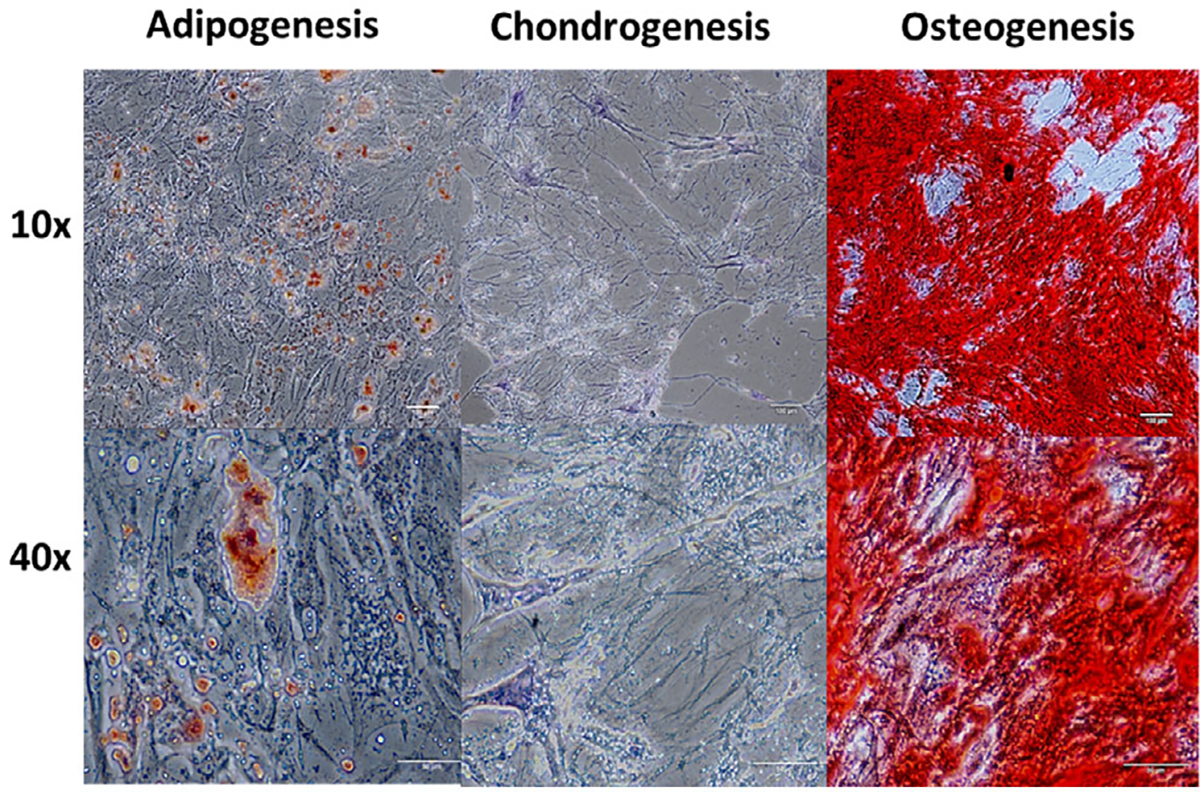
MSCs were removed from zein scaffolds after 7 days in culture in general media (GM) followed by 21 days on TCP in induction media for adipogenesis, chondrogenesis, or osteogenesis. Oil Red O staining for lipid droplets (red) for cultures undergoing adipogenesis. Toluidine blue staining for proteoglycans (purple) for cultures undergoing chondrogenesis. Alizarin red staining for calcium deposition (red) for cultures undergoing osteogenesis. 10x and 40x magnification.

To investigate whether MSCs cultured on zein scaffolds maintained their multipotent capability, cells were removed from scaffolds after 7 days and cultured on TCP for 21 days. Lipid droplets were seen for MSCs undergoing adipogenesis (Figure 9a). Proteoglycans were present for cells undergoing chondrogenesis (Figure 9b) and mineralization was detected for cells undergoing osteogenesis (Figure 9c). Comparisons were made with positive and negative controls, cells maintained on TCP only for all lineages (Figure S4–S6, Supporting Information).

## 3. Discussion

Plant-derived zein scaffolds were compared with animal-derived that MSCs grew on fibrous zein scaffolds, attached, showing gelatin scaffolds for supporting MSC growth, adhesion, and infiltration into the scaffold. Hydrolytically stable scaffolds were formed using an epoxide crosslinker and studies demonstrated integrin expression, and had greater infiltration into zein scaffolds as compared to gelatin scaffolds. In addition, zein scaffolds maintained MSC multipotency and supported vascularization and cell growth in vivo, demonstrating fibrous zein scaffolds may be favorable as a scaffold for tissue engineering.

Zein cross-linked with TMPGE formed a more hydrolytically stable scaffold than gelatin as noted by the significantly higher swelling for gelatin as compared to zein scaffolds. These results can be attributed to the differences in amino acids between gelatin and zein. Gelatin contains hydrophilic amino acids^[18]^ whereas zein is composed of hydrophobic amino acids resulting in its relative insolubility in aqueous conditions.^[5]^ Deng et al. demonstrated uncross-linked electrospun zein immersed in water resulted in formation of aggregates and loss of fibrous morphology whereas uncross-linked electrospun gelatin scaffolds immediately dissolved.^[19]^ Instability of gelatin in aqueous conditions results from swelling and uptake of water, whereas for zein, water acts as a plasticizer that results in shrinking or loss of fibrous morphology to lower surface energy.^[20]^ As demonstrated in these studies, zein scaffolds also reduced in dimension (e.g., diameter and thickness). Other groups have also observed the shrinking of zein scaffolds in aqueous conditions.^[21]^ Overall, both cross-linked gelatin and zein scaffolds were stable over the 21 days in hydrated conditions where TMPGE, an epoxy, can also be a viable crosslinker for gelatin scaffolds with minimal postprocessing. Other epoxy crosslinkers such as ethylene glycol diglycidyl ether have demonstrated stable gelatin films, but studies were not carried out long term.^[22]^ Crosslinkers such as glutaraldehyde, genipin, *N*-(3-dimethylaminopropyl)-*N*^0^-ethylcarbodiimide hydrochloride (EDC), and EDC with *N*-hydroxysulfosuccinimide (EDC-NHS) have been used to crosslink gelatin and/or collagen. EDC-NHS has demonstrated long-term stability for gelatin,^[23]^ however, EDC-NHS requires long postprocessing and immersion of electrospun mats in ethanolic conditions to crosslink, which can disturb fiber morphology and can dissolve zein.^[23]^ Glutaraldehyde and citric acid have also been utilized to crosslink zein films but may have issues with cytotoxicity, in the case of glutaraldehyde, and citric acid crosslinkers have not been evaluated long term.^[12]^ Crosslinking with TMPGE requires minimal postprocessing and the crosslinker can be incorporated directly into the electrospinning solution, which is advantageous for preserving fiber morphology and scaffold integrity.

Cell growth and adhesion were favorable for MSCs seeded on zein scaffolds. Cell number was comparable for MSCs seeded on zein scaffolds without serum compared to that of gelatin scaffolds without serum suggesting that zein can be a suitable substrate for MSCs even without the presence of serum. At 24 h, both zein and gelatin groups with serum had higher cell number and were more spread compared to groups without serum, but no significant differences were observed in serum-free conditions when comparing 1 and 24 h for all groups, complementing studies performed on zein films.^[13]^ Studies involving MSC attachment in serum-free conditions on TCP, gelatin, and fibronectin have been conducted.^[24]^ They demonstrated that MSCs seeded on gelatin and fibronectin are viable substrates in serum-free conditions, unlike MSCs on TCP substrates in the absence of serum, demonstrating their potential for use as substrates for MSC expansion. These results suggest zein is conducive for MSC attachment even without serum. The addition of serum facilitates greater cell attachment and spreading, suggesting protein adsorption occurs readily on zein.

Cell attachment was further investigated in this study where integrins and FAK were observed for cells on zein in conditions without serum. However, some differences in gene expression existed between zein and gelatin. Gene expression of PTK2, which encodes for FAK, a tyrosine kinase involved in integrinmediated signal transduction,^[25]^ was comparable across groups at 1 h suggesting that MSCs were attached even in serum-free conditions. Gelatin and zein are composed of amino acids which contain specific cell-binding amino acid sequences. RGD is a cell-binding sequence found in gelatin and other ECM adhesion proteins such as fibronectin^[16]^ while the LDV sequence is found in zein and other ECM adhesion proteins such as fibronectin.^[13]^ Integrins that bind to RGD include α_V_β_3_^[16]^ while integrins that bind to LDV are α_4_β_1_.^[26]^ Gene expression for ITGAV, the gene that encodes α_V_, and ITGB3, the gene that encodes β_3_, was determined for both gelatin and zein groups; however, expression was higher for gelatin at 1 and 24 h, respectively, indicating the relative importance of α_V_ and β_3_ integrins for attaching to gelatin in serum-free condition.^[27]^ Whereas cells had higher gene expression for ITGB1, the gene that encodes for β_1_, and ITGA4, the gene that encodes for α_4_, for the zein group at 1 h in conditions without serum, suggesting the relative importance of β_4_ and β_1_ integrins when attaching to zein and that cells could be binding to zein via the LDV sequence. However, further studies are necessary since cells can also secrete ECM adhesion proteins such as fibronectin in serum-deprived conditions, thus facilitating cellmatrix adhesion.^[28,29]^ Several studies have demonstrated the expression of α_V_, β_3_, β_1,_ and α_4_ attaching to gelatin scaffolds;^[16,30]^ however, this study, to our knowledge, is the first demonstrating integrin expression for cells attached to zein scaffolds. Other studies have evaluated fibroblast adhesion on zein films showing transglutaminase expression, which may facilitate the attachment of fibroblasts to zein substrates by functioning as a bridge between integrins and matrix proteins.^[13]^ MSCs also express transglutaminase^[31]^ and may be using it to mediate attachment via fibronectin. Cells also infiltrated the zein scaffolds at a greater depth than the gelatin scaffolds, which may be due to enhanced migration on zein due to relative expression of integrins and FAK.^[32]^. Infiltration of cells in scaffolds is beneficial for tissue engineering applications to facilitate integration.^[33]^

Zein scaffolds loaded with or without MSCs also demonstrated vascularization, cell growth, and biocompatibility when implanted subcutaneously. Zein fibrous implants cross-linked with TMPGE supported angiogenesis as demonstrated by the presence of CD31 where angiogenesis is important for nutrient transport and healing^[34,35]^ and is consistent with findings using porous 3D zein scaffolds prepared with the solvent cast-porogen leaching method.^[10]^ In addition, we established cell growth in the zein fibrous implants using the Ki67 marker where both host cells and implanted MSCs may be proliferating as the cell-loaded implants had greater Ki67 than the cell-free implants, suggesting that zein may be a suitable carrier for MSCs. The fibrous architecture can provide improved cell attachment and growth due to greater surface area to volume ratio.^[36]^ In vivo, the fibrous structure was still observed at 6 weeks. Future studies will need to quantify the rate of degradation in vivo. Overall, biocompatibility of fibrous zein scaffolds was demonstrated with no pronounced appearance of giant cells, macrophages, or fibroblasts surrounding or within the scaffold over the course of 6 weeks.^[37,38]^ Future studies may be needed to further establish the biocompatibility of the TMPGE cross-linked zein in vivo since this study was performed in immunocompromised mice. While SCID mouse models are commonly used for cell therapies with or without biomaterials,^[39]^ there are limitations associated with these models in demonstrating biocompatibility. Specifically, since they lack B and T lymphocytes, the full immune response against zein implants such as the presence of antibodies against zein was not established although zein is currently used as coatings and as an excipient in pharmaceutical applications. Future studies may be needed, such as measuring the apoptosis of MSCs, to assess the toxicological risk of the cross-linked zein scaffold.

Studies also demonstrate that zein scaffolds hold promise as a potential substrate for expanding MSCs since the multipotent capability of MSCs was retained after culturing on zein scaffolds. Further evaluation of stem cell markers, such as Oct-4 or Sox-2,^[40]^ may be needed but the differentiation capabilities were established particularly with adipogenic and osteogenic markers. A lesser intense proteoglycan staining occurred for chondrogenesis, which may have been due to the low seeding density cultures utilized and standard TCP plates, where chondrogenesis is typically performed in a high density, micromass/pellet culture.^[41]^ Findings suggest that zein scaffolds may be utilized as substrates to culture MSCs instead of 2D TCP for improved survival and MSC function. These current studies along with the cell adhesion studies conducted in serum-free conditions may provide the basis to potentially utilize zein as a plant-derived substrate for MSC expansion clinically without the use of FBS, as the regulatory guidelines are encouraging xeno-free alternatives.^[42]^ As a result, zein could be a potential plant-derived alternative substrate for MSC expansion.

## 4. Conclusion

Fibrous zein scaffolds are supportive for cell growth, adhesion, and infiltration and may be a suitable alternative to animalderived gelatin as a scaffold for tissue engineering. In vivo studies demonstrated fibrous zein scaffolds promoted blood vessel formation and cell proliferation, which are precursors for tissue formation and suggest they are viable scaffolds/carriers for MSCs. Cells adhered to zein expressing integrins and FAK. In addition, the multipotent capability of MSCs was maintained on zein scaffolds, potentially providing a substrate for expansion of MSCs which may have utility clinically for cell manufacturing. Overall, these results demonstrate fibrous zein as a promising biomaterial for tissue regeneration applications.

## 5. Experimental Section

### Fabrication of Scaffolds:

Zein scaffolds were fabricated using previously described methods.^[15]^ Briefly, 30% (w/w) of purified zein (Fisher Scientific, Pittsburgh, PA, USA) was dissolved in 80/20 denatured ethanol (Fisher Scientific)/deionized water (DI) water and stirred at room temperature with magnetic stirring for 1.5 h. Gelatin scaffolds were fabricated using a modified version of previously described methods.^[43,44]^ Briefly, 24% (w/w) bovine gelatin type B (Sigma Aldrich) was mixed in 60/40 glacial acetic acid (Fisher Scientific) and DI water which were stirred and heated to 60 °C for 10 min prior to the addition of gelatin. The solution containing gelatin was then stirred for 1.5 h. For both zein and gelatin, 10% (w/w) TMPGE (Sigma Aldrich) crosslinker was added to the gelatin solution and stirred for 15 min prior to electrospinning. The electrospinning setup for zein consisted of a syringe pump, flow rate of 2–2.5 mL h^−1^, 18 G needle, voltage of 17–20 kV, and distance of 15–20 cm. The electrospinning setup for gelatin consisted of a syringe pump, flow rate of 3–3.5 mL h^−1^, voltage of 35–40 kv, 18 G needle, and distance of 30 cm. After electrospinning, zein and gelatin scaffolds containing 10% TMPGE were cured for 5.5 h with a starting temperature of 110 °C with a stepwise increase of 10 °C every 1 h until 150 °C or cured for 2 h at 120 °C, respectively. Scaffolds were stored in dry conditions until further use.

### Scaffold Characterization:

Cross-linked scaffolds were characterized for fiber morphology by SEM (JSM-7900 F JEOL, Peabody, MA, USA) by assessing fiber diameter and interfiber spacing. Scaffolds were sputter coated (EMS 150, TES, Hatfield, PA, USA) with gold-palladium and then viewed by SEM using an accelerating voltage of 2 kV and a working distance of 6.0 mm. The diameters and interfiber spacing of 20 fibers, 10 each from two different samples were measured by Image J ((National Institutes of Health (NIH), Bethesda, MD, USA) software, as previously reported.^[23]^

The Young’s modulus of the cross-linked scaffolds (n = 6) was determined by tensile testing using an Instron (Instron Single Column Testing System Model 3342, Instron Corporation, Norwood, MA, USA), as previously described.^[15]^ The scaffolds were hydrated in PBS (phosphate buffered saline, Fisher) for 2 h and then cut into 15 mm × 5 mm rectangular strips. Gauge length of 5 mm at 5 mm min^−1^ crosshead speed was utilized for tensile testing.

The hydrolytic stability measurements were evaluated by measuring percent swelling, fold change in diameter, and thickness of the scaffolds. Scaffolds were cut into 6 mm discs and weight, diameter, and thickness were measured. The scaffolds (*n* = 4) were then immersed in PBS at 37 °C. Weight, diameter, and thickness changes of the scaffolds were measured at time points: 0.16, 1, 6, 24, 96, 168, 336, and 504 h. PBS changes occurred every 3–4 days. Change in swelling was calculated using Equation (1)

$$\text{Swelling}\;\left(\text{\%}\right)=\frac{W_{\text{hydrated}\;}-W_{\text{intial}\;}}{W_{\text{initial}\;}}\times 100$$

where W_hydrated_ is the weight of the hydrated scaffold at each time point and W_initial_ is the initial dry weight of each scaffold as previously described.^[45]^ Changes in diameter and thickness at each time point were reported as a fold change compared to the initial dry diameter or thickness of each scaffold. Protein loss experiments of the scaffolds were conducted in parallel. The PBS (*n* = 4) from the immersed samples was collected and analyzed for the amount of protein present in the supernatant. This was determined using the Pierce Bicinchoninic acid (BCA) Protein Assay Kit (Fisher) using standards for gelatin and zein. Percent protein loss was determined by normalizing the mass of protein in the supernatant to the initial mass of protein in the scaffold (prior to immersion) × 100. Time points measured were 0.16, 1, 24, 72, 168, 504, 672, 840, and 1008 h.

### In Vitro Cell Culture Studies:

Human MSCs were isolated and characterized from human bone marrow aspirates (Lonza, Walkersville, MD, USA), from male and female donors, 18–29 years of age, following previously published protocols.^[46]^ Cryopreserved, passage 3 MSCs were thawed and expanded in GM consisting of Dubelcco’s Modified Eagle’s Medium (DMEM, Gibco, Carlsbad, CA, USA), 10% fetal bovine serum (FBS, Hyclone, Logan, UT, USA), and 1% 100× antibiotic antimycotic (Gibco, Carlsbad, CA, USA) until 70–80% confluent. MSCs at passage 4 were seeded onto 10% TMPGE cross-linked gelatin and zein scaffolds. All scaffolds were sterilized in 100% ethanol (Fisher), washed with PBS, and then immersed in GM overnight until cell seeding the next day. Biopsy punches (Integra, Plainsboro, New Jersey, USA) were used to produce 6 mm diameter scaffold disks for gelatin and 7 mm diameter disks for zein because zein scaffolds shrink after sterilization and treatment in GM overnight due to their inherent hydrophobic properties. The 6 mm diameter scaffold disks (gelatin) and 7 mm diameter scaffold disks (zein), with thicknesses ranging from 0.28 to 0.30 mm, were placed in low attachment plates (96 well plates, nontreated, Fisher) while 12 mm scaffold disks were placed in low attachment plates (24 well plates, ultra-low attachment, Fisher). Tissue culture-treated polystyrene plates (TCP, Fisher) were used as controls. Cells were seeded at a density of 30 000 cells cm^−2^ on scaffolds and TCP controls. All cultures were maintained at 37 °C in humidified 5% CO_2_ incubators. Cell culture medium was changed every 3–4 days and harvested at time points stated.

### Cell Growth and Viability Studies:

Cell number over time was determined using the PicoGreen (Invitrogen, Waltham, MA, USA) assay kit. Briefly, cells were lysed in 0.1% triton x-100 (Sigma). The cell lysates and standards of known cell numbers were then treated with the PicoGreen reagent. Fluorescent intensities were measured at 480 nm/520 nm excitation/emission using a fluorescence microplate reader (FLX800, BioTek Instruments Inc., Winooski, VT, USA). Cell number was determined using a standard curve of known cell numbers. Cell number was evaluated at 1, 4, 7, 11, and 14 days with a sample size of *n* = 5 per group per time point.

Cell viability of the cross-linked scaffolds was determined by the CellTiter-Glo Luminescent Cell Viability Assay (Promega Corporation, Madison, WI, USA). The assay was conducted following manufacturer’s protocols. Briefly, at each time point, CellTiter-Glo reagent was added to each lysed sample and read using the fluorescent microplate reader (FLX800, BioTek Instruments Inc.). The amount of ATP for each sample was calculated based on an ATP standard curve using an ATP standard (Ultra-Pure ATP, 100 mM, Promega). MSC morphology was observed by actin staining using rhodamine phalloidin (Invitrogen). Samples were fixed with 4% paraformaldehyde (Sigma) for 20 min, treated with 0.1% Triton X in PBS for 15 min, and treated with Rhodamine phalloidin (1:100) for 1 h. The cells were viewed on days 1, 4, 7, and 14 using confocal microscopy (C1si Confocal Microscope, Nikon Instruments Inc., Melville, NY, USA). Cell viability was also assessed with the LIVE/DEAD viability/cytotoxicity kit (Invitrogen) to identify live and dead cells. Cells were treated with the live/dead stain (green, fluorescent calcein-AM, and red-fluorescent ethidium homodimer-1 (EthD-1)) for 30 min. The cells were viewed at 1, 7, and 14 days using confocal microscopy. All experiments were repeated at least twice.

### Cell Adhesion Studies:

For cell adhesion studies, MSCs were either seeded on scaffolds in GM as previously described or in GM without 10% serum (GM-0% serum, 1% antibiotic-antimycotic), and harvested at 1 and 24 h. Seeding density of 30 000 cells cm^−2^ (10 000 cells per scaffold) was used for scaffolds and 9000 cells cm^−2^ (3000 cells per well) was used for TCP at 1 and 24 h. A higher seeding density was used on the scaffolds as compared to TCP to obtain similar numbers of cells attached at 1 h. Cell number was also evaluated using PicoGreen, as described in Section 2.4. Immunostaining of adhesion markers FAK (monoclonal mouse anti-human, Invitrogen), αvβ_3_ (monoclonal mouse anti-human, eBioscience, San Diego, CA, USA), β_1_ (polyclonal rabbit anti-human, Invitrogen), and α_4_ (monoclonal mouse anti-human, eBioscience) integrins was performed. The cells seeded on scaffolds were fixed and permeabilized as described above. Samples were then blocked with SuperBlock (TBS) Blocking Buffer Dry Blend (Thermo Fisher Scientific, Waltham, MA, USA) for 1 h. The primary antibody was added and left overnight at 4 °C. After washing with PBS, the secondary antibodies either Alexa Fluor 561 goat anti-mouse for FAK (Invitrogen), Alexa Fluor 488 goat anti-mouse for αvβ_3_, α_4_ (Invitrogen) or Alexa Fluor 561 donkey anti-rabbit for β_1_ (Invitrogen) were added, and samples were incubated for 1 h. The cells were then viewed using confocal microscopy (C1si Confocal Microscope).

Gene expression was conducted using quantitative reverse transcriptase-polymerase chain reaction (RT-PCR) for MSCs seeded on the scaffolds and TCP in conditions with or without serum. Briefly, samples were harvested at 1 and 24 h. RNA was isolated using TRIzol (Invitrogen) extraction and purified using the RNeasy micro kit (Qiagen, Valencia, CA, USA) according to manufacturer’s protocol including DNase digestion step. RT-PCR was conducted using the One Step QuantiTect SYBR Green RT-PR kit (Qiagen) and the MX3000P qPCR system (Agilent Technologies, Santa Clara, CA, USA) according to previously published protocols.^[47]^ Data were normalized to the housekeeping gene RPLPO. The following adhesion markers were examined: protein kinase 2 (PTK2), integrin subunit alpha V (IGTAV), integrin subunit beta 3 (ITGB3), integrin subunit beta 1 (ITGB1), and integrin subunit alpha 4 (IGTA4). Hs_PTK2_1_SG, Hs_ITGAV_1_SG, Hs_ITGB3_1_SG, Hs_ITGA4, and Hs_ITGB1_1_SG QuantiTect Primer Assays were purchased from Qiagen. Samples were run in triplicate.

### Cell Penetration into the Scaffolds:

MSC infiltration or penetration into the scaffolds was investigated by obtaining z-stack images using confocal microscopy (C1si Confocal Microscope). All images were obtained within the range of 0–240 μm (thickness of the scaffolds). The 3D volume viewer function using the NIS Elements software (Nikon) was used to visualize 3D projections of the cells within the scaffolds. The average depth penetration was quantified by evaluating the z slices at every 10 μm to determine the largest penetration depth of cells at each field of view. Scaffolds, *n* = 3 per group at 1 h and *n* = 8 per group at 1 day, were imaged using four different areas or fields of view per group. A total sample size of *n* = 14 (gelatin and zein) at 1 h and *n* = 32 (zein) and *n* = 28 (gelatin) were evaluated at 1 day.

### In Vivo Biocompatibility in an Ectopic Model:

Implants were prepared by electrospinning a 40% (w/w) of zein in 80/20 ethanol/DI water solutions with 10% TMPGE solution, followed by curing as described above. To create large pores for blood vessel ingrowth into the scaffolds, a custom needle-array containing 30 G needles was utilized. Scaffolds were immersed in liquid nitrogen and a needle array was inserted into the scaffold to create the pores. Scaffolds were then cut to the following dimensions: 5 mm × 5 mm × 1 mm for implantation. Scaffolds were sterilized using gamma irradiation and stored at room temperature until further use.

### Zein Implant Characterization:

Fiber diameter and interfiber spacing measurements were conducted as described above. Pore diameter was measured using Image J for n = 10 measurements for two different implants per group.

### Cell-Loaded Implants:

Human MSCs passage 3 from two different donors (male and female) were mixed at a 50:50 ratio^[48]^ to reduce variability in this initial study. They were loaded onto implants at a cell density of 5 000 000 cells mL^−1^ using a vacuum loading technique using methods as previously described.^[49,50]^ Six implants per group were used. Cell-laden scaffolds in 5 mL Falcon tubes (Fisher) in GM were placed in the incubator overnight at 37 °C, 5% CO_2_ to allow for cell attachment. On the day of the surgery, tubes containing the cell-laden scaffolds were placed on ice at 4 °C until implantation. Cell-free scaffolds were used as a control.

### In Vivo Study:

All animal procedures were in accordance with an approved animal protocol by the Rutgers University Institutional Animal Care and Use Committee (IACUC, Protocol ID: 202 200 254). Implants were placed subcutaneously in the backs of C.B.17 SCID mice (male, 12–16 week-old and 25–30 g). MSC-loaded implants and cell-free implants (n = 6 per group) were inserted subcutaneously.^[49]^ Briefly, a 1 cm transverse incision was made along the dorsum of the back. Blunt scissors were used to create six pockets under the skin and the implants were inserted. The position of cell-loaded and cell-free implants was randomized per mouse. The wound was closed using stainless steel skin staples (MikRon Precision, Inc. Gardena, CA, USA). Implants were harvested at 2 and 6 weeks. Implants were fixed in 4% paraformaldehyde for 24 h at room temperature, then stored in 70% ethanol until sectioning.

### Histological Analysis:

The implants were processed for routine paraffin embedding, sectioned and stained with H&E and Masson’s trichrome staining at 2 and 6 weeks. Routine immunohistochemistry was conducted to stain for CD31 (PECAM-1 specific to mouse) (D8V9E) (Cell Signaling Technologies, Danvers, MA, USA), a marker for vascularization, and Ki67 (reacts with both mouse and human, SP6-ab16667) (Abcam, Fremont, CA, USA), a proliferation marker, at 2 weeks. All sectioning and staining were conducted at the Digital Imaging and Histology Core, Rutgers-NJMS Cancer Center. Percent area stained with CD31 was calculated by selecting region of interest and averaging areas for two different 4× images per sample for n = 5 or 6 samples per group. Thresholding in the Image J software was utilized to first isolate CD31 stained samples and then percent area was calculated using the software.^[51,52]^ Similarly, for quantifying Ki67, percent area of Ki67 positive cells was measured and averaged for five different 40× images per sample for *n* = 6 samples per group. The thresholding function was utilized to calculate percent area for Ki67.

### Multipotent Capability of Cells Seeded on Zein Scaffolds:

Trilineage capability of MSCs seeded on zein scaffolds was determined by culturing MSCs on zein scaffolds and compared with cells cultured on TCP for 7 days. After 7 days, cells were trypsinized. For TCP groups cells were incubated in trypsin for 5 min. For scaffolds groups, cells were incubated in trypsin for 30 min. Cells were resuspended in GM and seeded at 6000 cells cm^−2^ on TCP. Cells were cultured for 21 days in medium to induce adipogenesis, chondrogenesis, or osteogenesis, according to previously reported protocols.^[53]^ Comparisons were made with positive controls (MSCs not grown on scaffolds and cultured in differentiation media) and negative controls (MSCs in GM). After 21 days, cultures were fixed in 4% paraformaldehyde and stained. Oil Red O stain (0.3% Oil Red O (Sigma) w/v in isopropanol) was used to stain lipid droplets. Toluidine blue (0.05% (wt/vol) toluidine blue (Sigma) in 0.2 m sodium phosphate 0.1 m citrate buffer pH 4.0) stains proteoglycans. Alizarin red (0.02% w/v solution of Alizarin red (Sigma) in DI water, pH 4.1–4.3) stains calcium. Images were taken at 4× and 40× with a Nikon Eclipse light microscope from a sample size of n = 2 and the study was repeated twice.

### Statistical Analysis:

All data were reported as mean ± standard deviation (S.D.), unless otherwise stated. One-way and two-way analysis of variance (ANOVA) were used for statistical analyses with Tukey’s post hoc tests to determine statistical significance between groups at *p* < 0.05. Independent sample’s t-test was used for statistical analysis between two independent groups to determine statistical significance at *p* < 0.05.

## Supplementary Material

Source Data File

Supplemental Figures

## Figures and Tables

**Table 1.** Fiber diameter, interfiber spacing, and young’s modulus of cross-linked gelatin and zein scaffolds.

Matrix characterization	Gelatin	Zein

Fiber diameter [μm]	1.25 ± 0.50	1.44 ± 0.55
Interfiber spacing [μm]	24.6 ± 10.8	26.2 ± 13.5
Young’s modulus [kPa]	194 ± 53	195 ± 47

## Data Availability

All of the data that support the findings of this study are contained within the main manuscript, supplementary materials, or are available at the following DOI https://doi.org/10.7916/tszp-9a27.

## References

[1] Chan BP, Leong KW, Eur. Spine J. 2008, 4, 467. 10.1007/s00586-008-0745-3 PMC2587658 19005702

[2] aBhattarai DP, Aguilar LE, Park CH, Kim CS, Membranes 2018, 8; 30110968 10.3390/membranes8030062 PMC6160934

[3] Iravani S, Varma RS, Green Chem. 2019, 21, 4839.

[4] Sun QS, Dong J, Lin ZX, Yang B, Wang JY, Biopolymers 2005, 78, 268. 15898117 10.1002/bip.20298

[5] Pérez-Guzmán CJ, Castro-Muñoz R, Processes 2020, 8, 1376.

[6] Tortorella S, Maturi M, Vetri Buratti V, Vozzolo G, Locatelli E, Sambri L, Comes Franchini M, RSC Adv. 2021, 11, 39004. 35492476 10.1039/d1ra07424e PMC9044754

[7] Ru J, Wei Q, Yang L, Qin J, Tang L, Wei J, Guo L, Niu Y, RSC Adv. 2018, 8, 18745. 35539669 10.1039/c8ra02595a PMC9080628

[8] Qu ZH, Wang HJ, Tang TT, Zhang XL, Wang JY, Dai KR, Acta Biomater. 2008, 4, 1360. 18439886 10.1016/j.actbio.2008.03.006

[9] Tu J, Wang H, Li H, Dai K, Wang J, Zhang X, Biomaterials 2009, 30, 4369. 19539987 10.1016/j.biomaterials.2009.04.054

[10] Wang HJ, Gong SJ, Lin ZX, Fu JX, Xue ST, Huang JC, Wang JY, Biomaterials 2007, 28, 3952. 17582490 10.1016/j.biomaterials.2007.05.017

[11] Zhang JF, Wang Y, Liao S, Lallier T, Wen ZT, Xu X, Oral Health Dent. Studies 2017, 1, 1. PMC6409179 30863833

[12] Reddy N, Reddy R, Jiang Q, Trends Biotechnol. 2015, 33, 362. 25887334 10.1016/j.tibtech.2015.03.008

[13] Cui H, Liu GL, Padua GW, Colloids Surf., B 2016, 145, 839. 10.1016/j.colsurfb.2016.05.048 27315332

[14] Vasita R, Katti DS, Int. J. Nanomed. 2006, 1, 15. 10.2147/nano.2006.1.1.15 PMC2426767 17722259

[15] Cardenas Turner J, Collins G, Blaber EA, Almeida EAC, Arinzeh TL, Tissue Eng J. Regener. Med. 2020, 14, 173. 10.1002/term.2984 31670902

[16] Davidenko N, Schuster CF, Bax DV, Farndale RW, Hamaia S, Best SM, Cameron RE, J. Mater. Sci. Mater. Med. 2016, 27, 148. 27582068 10.1007/s10856-016-5763-9 PMC5007264

[17] Mohan N, Detamore MS, in Nanotechnology Applications For Tissue Engineering, (Eds: Thomas S, Grohens Y, Ninan N), William Andrew Publishing, Oxford 2015.

[18] Campiglio CE, Contessi Negrini N, Farè S, Draghi L, Materials 2019, 12, 2476. 31382665 10.3390/ma12152476 PMC6695673

[19] Deng L, Kang X, Liu Y, Feng F, Zhang H, Food Hydrocolloids 2018, 74, 324.

[20] Xu W, Karst D, Yang W, Yang Y, Polym. Int. 2008, 57, 1110.

[21] Alhusein N, Blagbrough IS, De Bank PA, Drug Delivery Transl. Res. 2013, 3, 542. 10.1007/s13346-013-0179-2 25786374

[22] Vargas G, Acevedo JL, López J, Romero J, Mater. Lett. 2008, 62, 3656.

[23] Huang GP, Shanmugasundaram S, Masih P, Pandya D, Amara S, Collins G, Arinzeh TL, J. Biomed. Mater. Res., Part A 2015, 103, 762. 10.1002/jbm.a.35222 24828818

[24] Jung S, Sen A, Rosenberg L, Behie LA, Cytotherapy 2010, 12, 637. 20608762 10.3109/14653249.2010.495113

[25] Geiger B, Bershadsky A, Cell 2002, 110, 139. 12150922 10.1016/s0092-8674(02)00831-0

[26] Clements JM, Newham P, Shepherd M, Gilbert R, Dudgeon TJ, Needham LA, Edwards RM, Berry L, Brass A, Humphries MJ,J.Cell Sci. 1994, 107, 2127. 7527054 10.1242/jcs.107.8.2127

[27] Lee JW, Kim YH, Park KD, Jee KS, Shin JW, Hahn SB, Biomaterials 2004, 25, 1901. 14738854 10.1016/j.biomaterials.2003.08.037

[28] Kleinman HK, Luckenbill-Edds L, Cannon FW, Sephel GC, Anal.Biochem. 1987, 166, 1. 3314585 10.1016/0003-2697(87)90538-0

[29] Grinnell F, Feld MK, Cell 1979, 17, 117. 378401 10.1016/0092-8674(79)90300-3

[30] Zaman MH, Biophys. J. 2007, 92, L17. 17098789 10.1529/biophysj.106.097519 PMC1751409

[31] Song H, Chang W, Lim S, Seo HS, Shim CY, Park S, Yoo KJ, Kim BS, Min BH, Lee H, Jang Y, Chung N, Hwang KC, Stem Cells 2007, 25, 1431. 17347495 10.1634/stemcells.2006-0467

[32] Hauck CR, Hsia DA, Schlaepfer DD, IUBMB Life 2002, 53, 115. 12049193 10.1080/15216540211470

[33] Moy J, Limaye A, Arinzeh TL, Artificial Protein And Peptide Nanofibers: Design, Fabrication, Characterization, and Applications, Woodhead Publishing, United Kingdom 2020, p. 351.

[34] Sivaraj KK, Adams RH, Development 2016, 143, 2706. 27486231 10.1242/dev.136861

[35] Beamer B, Hettrich C, Lane J, HSS J 2010, 6, 85. 19763695 10.1007/s11420-009-9129-4 PMC2821499

[36] Stevens MM, George JH, Science 2005, 310, 1135. 16293749 10.1126/science.1106587

[37] Kyriakides TR, Kim H-J, Zheng C, Harkins L, Tao W, Deschenes E, Biomed. Mater. 2022, 17, 022007. 10.1088/1748-605X/ac5574 PMC9159526 35168213

[38] Yang J, Jao B, McNally AK, Anderson JM, J. Biomed. Mater. Res, Part A 2014, 102, 2017. 10.1002/jbm.a.35152 24616384

[39] Costa-Pinto A, Santos TC, Neves NM, Reis RL, in Biomaterials from Nature for Advanced Devices and Therapies, John Wiley & Sons, New Jersey 2016.

[40] Matic I, Antunovic M, Brkic S, Josipovic P, Mihalic KC, Karlak I, Ivkovic A, Marijanovic I, Open Access Maced. J. Med. Sci. 2016, 4, 9. 27275321 10.3889/oamjms.2016.008 PMC4884261

[41] Mackay AM, Beck SC, Murphy JM, Barry FP, Chichester CO, Pittenger MF, Tissue Eng. 1998, 4, 415. 9916173 10.1089/ten.1998.4.415

[42] Cimino M, Gonçalves RM, Barrias CC, Martins MCL, Stem Cells Int. 2017, 2017, 6597815. 29158740 10.1155/2017/6597815 PMC5660800

[43] Erencia M, Cano F, Tornero JA, Fernandes MM, Tzanov T, Macanás J, Carrillo F, J. Appl. Polym. Sci. 2015, 132, 42115.

[44] Huang GP, Menezes R, Vincent R, Hammond W, Rizio L, Collins G, Arinzeh TL, Tissue Eng., Part A 2017, 23, 1011. 28285569 10.1089/ten.TEA.2016.0461

[45] Menezes R, Arinzeh TL, Ann. Biomed. Eng. 2020, 48, 2040. 32285342 10.1007/s10439-020-02499-9

[46] Haynesworth SE, Goshima J, Goldberg VM, Caplan AI, Bone 1992, 13, 81. 1581112 10.1016/8756-3282(92)90364-3

[47] Shanmugasundaram S, Chaudhry H, Arinzeh TL, Tissue Eng., Part A 2011, 17, 831. 20973751 10.1089/ten.TEA.2010.0409

[48] Widholz B, Tsitlakidis S, Reible B, Moghaddam A, Westhauser F, Cells 2019, 8, 633. 31238494 10.3390/cells8060633 PMC6628337

[49] Arinzeh TL, Tran T, McAlary J, Daculsi G, Biomaterials 2005, 26, 3631. 15621253 10.1016/j.biomaterials.2004.09.035

[50] Damaraju SM, Shen Y, Elele E, Khusid B, Eshghinejad A, Li J, Jaffe M, Arinzeh TL, Biomaterials 2017, 149, 51. 28992510 10.1016/j.biomaterials.2017.09.024

[51] Schneider CA, Rasband WS, Eliceiri KW, Nat. Methods 2012, 9, 671. 22930834 10.1038/nmeth.2089 PMC5554542

[52] Hunter JM, Kwan J, Malek-Ahmadi M, Maarouf CL, Kokjohn TA, Belden C, Sabbagh MN, Beach TG, Roher AE, PloS One 2012, 7, e36893. 22615835 10.1371/journal.pone.0036893 PMC3353981

[53] Pittenger MF, Mackay AM, Beck SC, Jaiswal RK, Douglas R, Mosca JD, Moorman MA, Simonetti DW, Craig S, Marshak DR, Science 1999, 284, 143. 10102814 10.1126/science.284.5411.143

