# Supplementary material for: Plant-Derived Zein as an Alternative to Animal-Derived Gelatin for Use as a Tissue Engineering Scaffold
Source: Adv Nanobiomed Res. Author manuscript; Available in PMC 2024 Apr 25. (PMC11045004; doi:10.1002/anbr.202300104)
Supplement: Supplemental Figures [file NIHMS1957581-supplement-Supplemental_Figures.pdf]

Supplementary Figures

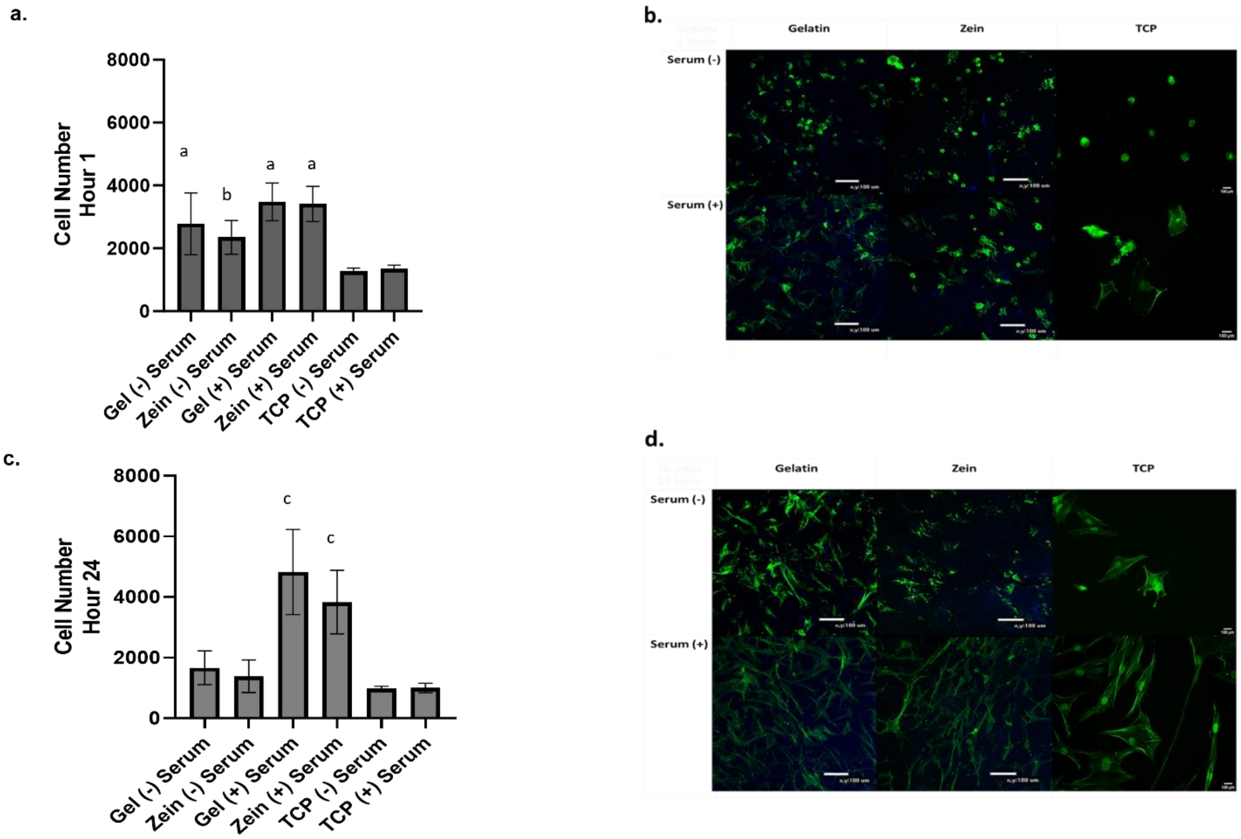

Figure S1 (a) Cell number at 1 hour for gelatin, zein and TCP groups with or without serum. **(b)** Confocal images of MSCs seeded on gelatin, zein and TCP with or without serum stained for actin (green) at 1 hour. **(c)** Cell number at 24 hours for gelatin, zein, and TCP groups with or without serum. **(d)** Confocal images of MSCs seeded on gelatin, zein and TCP with or without serum stained for actin (green) at 24 hours. [a]$p<0.05$ is significantly different from TCP (-) Serum and TCP (+) Serum, [b]$p<0.05$ is significantly different from Gel (+) Serum, [c]$p<0.05$ is significantly different from all other groups.

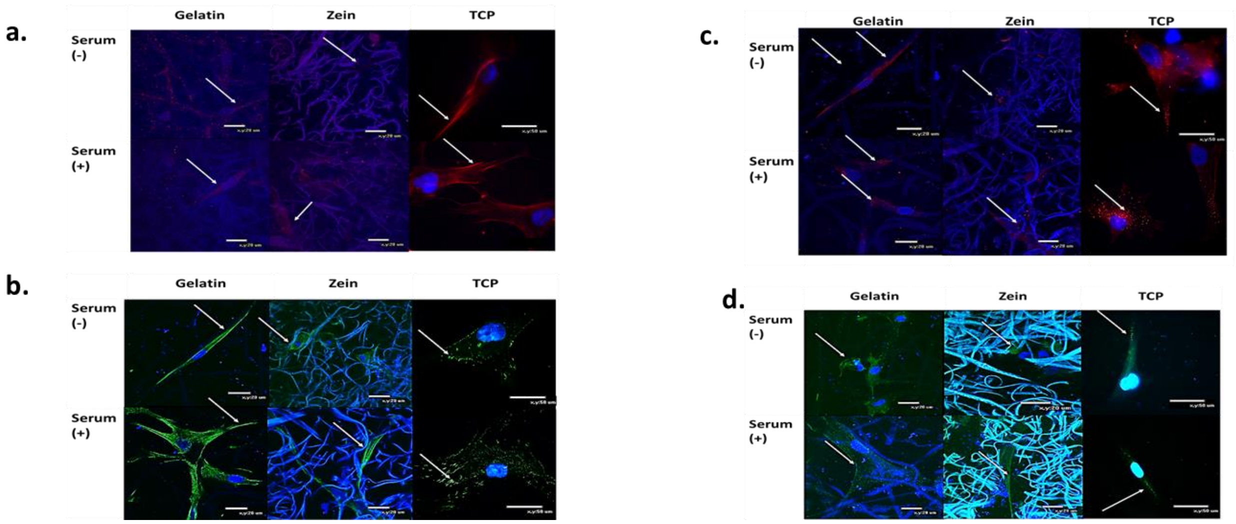

Figure S2. Immunostaining for (a) FAK (red), (b) αvβ3 (green), (c) β1 (red) and (d) α4 (green) for cells on gelatin, zein and TCP at 24 hours. Nucleus is stained blue. Scale bar: 20 μm for scaffolds and 50μm for TCP.

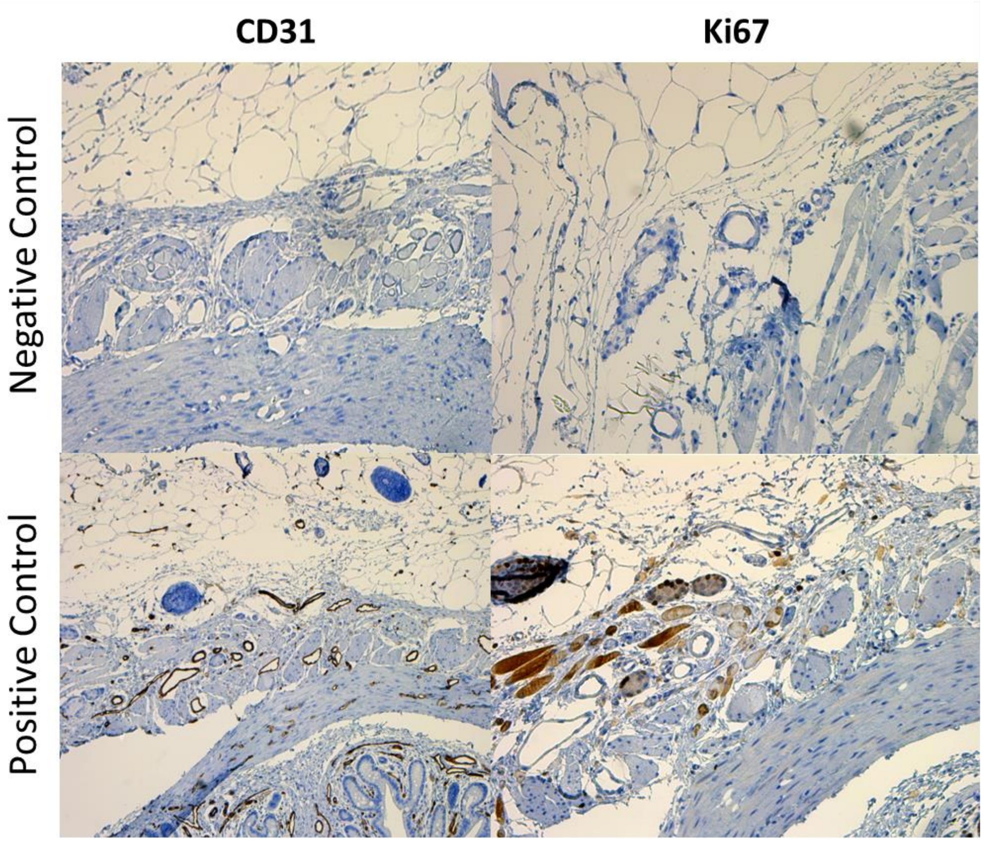

Figure S3. Positive and negative controls of immunostaining for CD31 and Ki67. Magnification 20x.

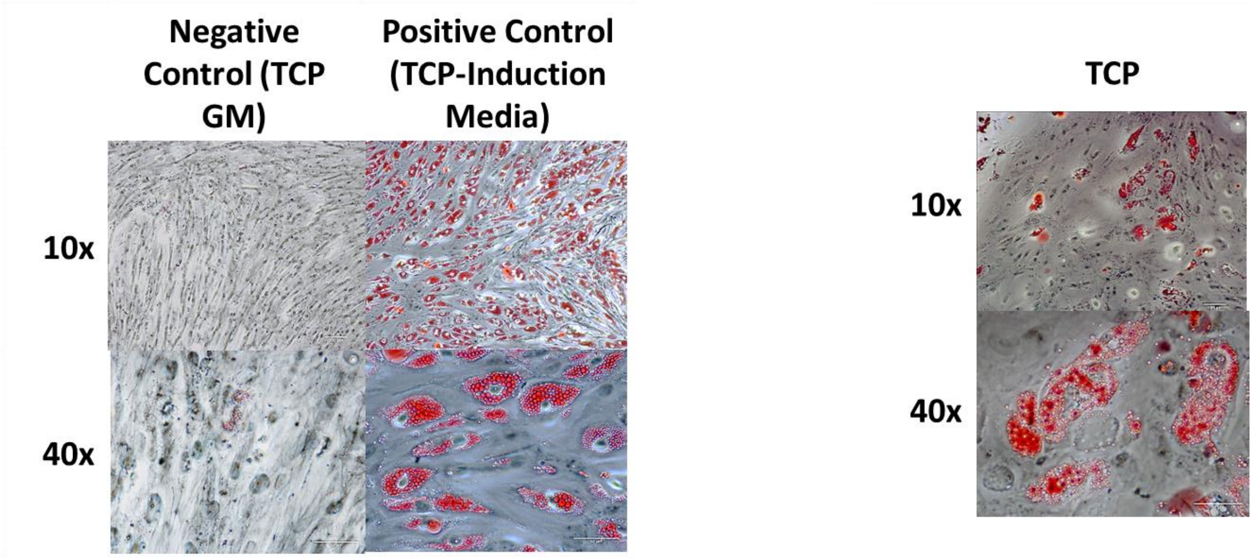

Figure S4. Oil Red O staining showing lipid droplets (red) for MSCs undergoing adipogenesis on TCP. (left panel) Negative control is MSCs cultured in growth media. Positive control is MSCs cultured in adipogenesis induction media. (right panel) Lipid droplets for MSCs detached from TCP and cultured on TCP substrates for 21 days in adipogenic induction media. 10x and 40x magnification.

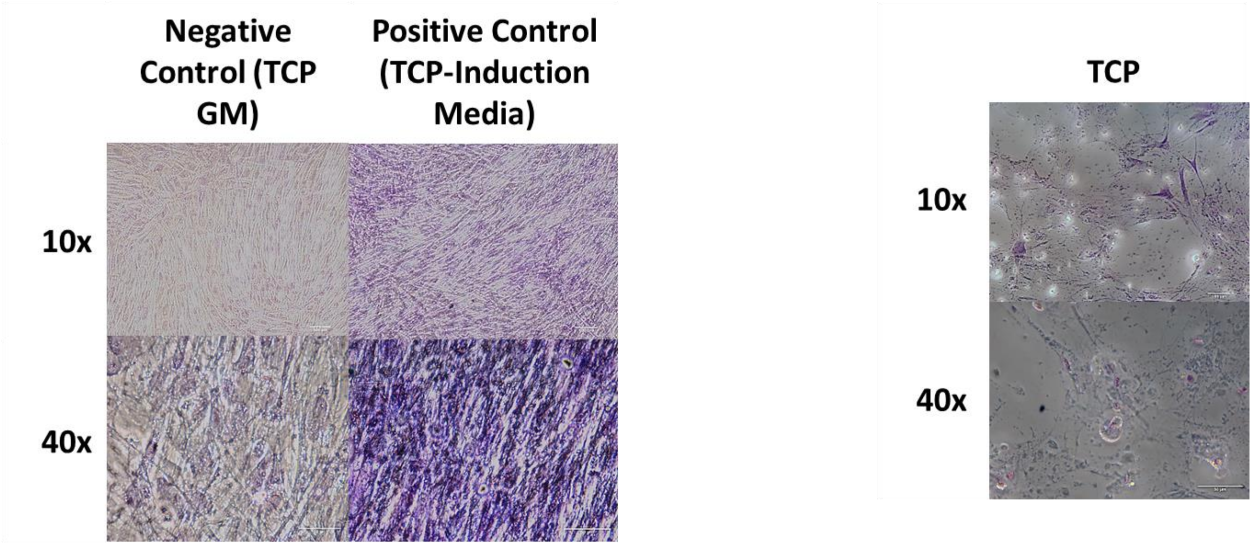

Figure S5. Toluidine blue staining for proteoglycans (purple) for MSCs undergoing chondrogenesis on TCP. (left panel) Negative control is MSCs cultured in growth media. Positive control is MSCs cultured in chondrogenic induction media. (right panel) Proteoglycans stained for MSCs detached from TCP and cultured on TCP substrates for 21 days in chondrogenic induction media. 10x and 40x magnification.

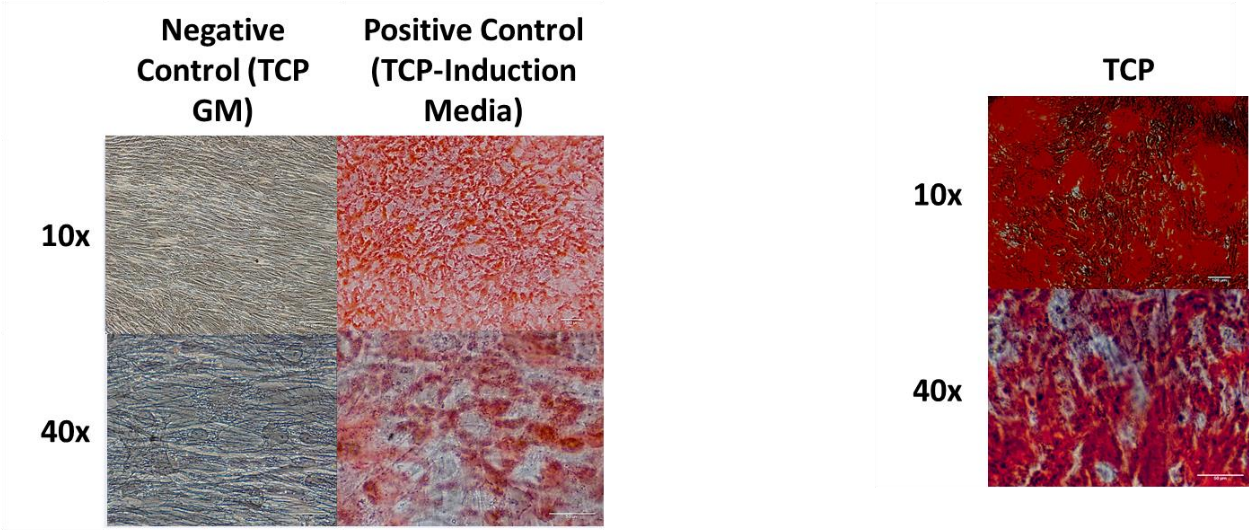

Figure S6. Alizarin red staining of calcium (red) deposited for MSCs undergoing osteogenesis on TCP. (left panel) Negative control is MSCs in growth media. Positive control is MSCs in osteogenic induction media. (right panel) Calcium deposition for MSCs detached from TCP and cultured on TCP substrates for 21 days in osteogenic induction media. 10x and 40x magnification.
